# Myorelaxant Effect of the *Dysphania ambrosioides* Essential Oil on *Sus scrofa domesticus* Coronary Artery and Its Toxicity in the *Drosophila melanogaster* Model

**DOI:** 10.3390/molecules26072041

**Published:** 2021-04-02

**Authors:** Luiz Jardelino de Lacerda Neto, Andreza Guedes Barbosa Ramos, Renata Evaristo Rodrigues da Silva, Luís Pereira-de-Morais, Fernanda Maria Silva, Roger Henrique Sousa da Costa, Lindaiane Bezerra Rodrigues Dantas, José Galberto Martins da Costa, Henrique Douglas Melo Coutinho, Grażyna Kowalska, Joanna Hawlena, Radosław Kowalski, Roseli Barbosa, Francisco Assis Bezerra da Cunha

**Affiliations:** 1 Department of Biological Chemistry, Regional University of Cariri, Crato 63105-000, Brazil; luizjardelino@gmail.com (L.J.d.L.N.); andrezaurca@gmail.com (A.G.B.R.); renata_ers@hotmail.com (R.E.R.d.S.); luispereira256@gmail.com (L.P.-d.-M.); fernandamsmv@gmail.com (F.M.S.); rogerhenrique8@hotmail.com (R.H.S.d.C.); lindaianebrd@gmail.com (L.B.R.D.); galberto.martins@gmail.com (J.G.M.d.C.); roselibarbo@gmail.com (R.B.); cunha.urca@gmail.com (F.A.B.d.C.); 2 Department of Tourism and Recreation, University of Life Sciences in Lublin, 15 Akademicka Street, 20-950 Lublin, Poland; grazyna.kowalska@up.lublin.pl (G.K.); hawlena@interia.pl (J.H.); 3 Department of Analysis and Evaluation of Food Quality, University of Life Sciences in Lublin, 8 Skromna Street, 20-704 Lublin, Poland

**Keywords:** essential oil, natural product, myorelaxative activity, toxicity

## Abstract

Purpose: Alternative methods for the use of animals in research have gained increasing importance, due to assessments evaluating the real need for their use and the development of legislation that regulates the subject. The principle of the 3R’s (replacement, reduction and refinement) has been an important reference, such that in vitro, ex vivo and cord replacement methods have achieved a prominent place in research. Methods: Therefore, due to successful results from studies developed with these methods, the present study aimed to evaluate the myorelaxant effect of the *Dysphania ambrosioides* essential oil (EODa) using a *Sus scrofa domesticus* coronary artery model, and the toxicity of both the *Dysphania ambrosioides* essential oil and its major constituent, α-terpinene, against *Drosophila melanogaster* in toxicity and negative geotaxis assays. Results: The EODa relaxed the smooth muscle of swine coronary arteries precontracted with K^+^ and 5-HT in assays using *Sus scrofa domesticus* coronary arteries. The toxicity results presented LC_50_ values of 1.546 mg/mL and 2.282 mg/mL for the EODa and α-terpinene, respectively, thus showing the EODa and α-terpinene presented toxicity to these dipterans, with the EODa being more toxic. Conclusions: Moreover, the results reveal the possibility of using the EODa in vascular disease studies since it promoted the relaxation of the Sus scrofa domesticus coronary smooth muscle.

## 1. Introduction

The use of animals in essential research has been constantly questioned in terms of the principles of the 3R’s (replacement, reduction and refinement), thus creating the need for reflection in the number of animals used in research (reduction), the possibility of replacing vertebrate animals with invertebrates, embryos, microorganisms, organs or isolated tissues, in addition to guaranteeing the quality of experiments (refinement), where this principle underscores training for the correct use of animal handling techniques, aseptic techniques in surgical procedures, correct dose administrations and other recommendations for carrying out high quality experiments [1].

The replacement of animals by in vitro and ex vivo methodologies efficiently promotes one of the R’s, avoiding the use of animals in assays, with the ability to clarify the toxic potential or the effectiveness of a substance or product under analysis [2]. In the 1990s, in vitro methodologies were practically unused to verify toxicity and efficacy, however, in the early 2000s a great growth in the use of this methodology to assess genotoxicity, pharmaceutical safety and pharmacokinetic assays was seen [3]. Another alternative method that is worth noting is the replacement of vertebrate animals by invertebrate ones in assays, which permit Parkinson’s disease, memory, endocrine, diabetes and toxicological assays to be studied. *Drosophila melanogaster* is a frequently used invertebrate, where this substitution is beneficial for a number of reasons such as cost reduction, short life cycle, small size and simple fly anatomy, in addition to being able to observe a larger number of animals per experiment [2]. *Drosophila melanogaster* is a model of great importance as it generates information for fighting pests and disease vectors. The search for new agents that are effective in the control of vectors, explore options that are economically viable, with lower environmental pollution and lower toxicity to non-target organisms, including humans [4].

In this context, the search for products with biological and/or pharmacological activity has reignited the interest in natural products, especially those with a plant origin, among which essential oils (EOs) occupy a prominent place. EOs are liquids at room temperature and are usually complex compounds formed by various substances such as alcohols, aldehydes, ketones, phenols, ethers, esters and terpenes at different proportions, with the latter being predominantly mono and sesquiterpenes. EOs possess antioxidant, metal chelation, free radical scavenging, pain-modulating, anticonvulsant, neuroprotective and anti-inflammatory activities [5,6].

*Dysphania ambrosioides* (L.), Mosyakin and Clemants (Chenopodiaceae), also known as *Chenopodium ambrosioides*, occurs frequently in subtropical and subtemperate regions with a cosmopolitan characteristic, and is popularly used for the treatment of worms [7], as a diuretic and in respiratory and inflammatory alterations. According to the world health organization [8], *D. ambrosioides* is one of the most commonly used medicinal plants in the world, this being due to its wide distribution and ethnomedical knowledge. The *D. ambrosioides* EO has previously been reported in the literature with insecticidal activity against bees and houseflies [9], as an antiviral, anthelmintic, antifungal antileishmanial and antioxidant. The EO possesses components with known medicinal value such as: ascaridol, isoascaridol, p-cymene, limonene and α-terpinene. Bioactive products isolated from plants are used for the treatment of tuberculosis, rheumatism, uterine hemorrhaging and respiratory diseases [10]. Studies have demonstrated α-terpinene as safe in embryo-fetotoxicity tests in rats [11], however, other studies have revealed some toxic effects in the oral use of this substance [12,13]. Therefore, among the concerns for the development of new products with biological and/or pharmacological activity is the determination of toxic doses and safety margins. Several studies demonstrate anatomical and physiological similarities between the cardiovascular system of humans and pigs (*Sus scrofa domesticus*), in particular the control of smooth muscle, with effects involving electromechanical and pharmacomechanical mechanisms being reported [14,15,16,17]. The myorelaxant effect that drugs and medications have on vascular smooth muscle is a fundamental tool in the control of conditions in which vasoconstriction or vascular obstruction are responsible for damage to tissues, organs, systems or even the survival of the organism. Therefore, tests with swine coronary arteries yield relevant information on the subject, as some studies have already demonstrated the myorelaxant action of extracts, essential oils and their components [18,19,20,21,22,23,24]. Thus, smooth muscle effects, particularly in coronary arteries, provide important information, which may generate enormous possibilities for other studies involving the action and mechanisms of contraction of smooth muscle. This study aimed to evaluate the myorelaxant activity of the *Dysphania ambrosioides* essential oil (EODa) in an ex vivo *Sus scrofa domesticus* coronary model, as well as to verify the toxic concentrations of the EODa and its major constituent, α-terpinene, against the *Drosophila melanogaster* alternative model.

## 2. Results

### 2.1. Phytochemical Analysis

**Figure 1:**
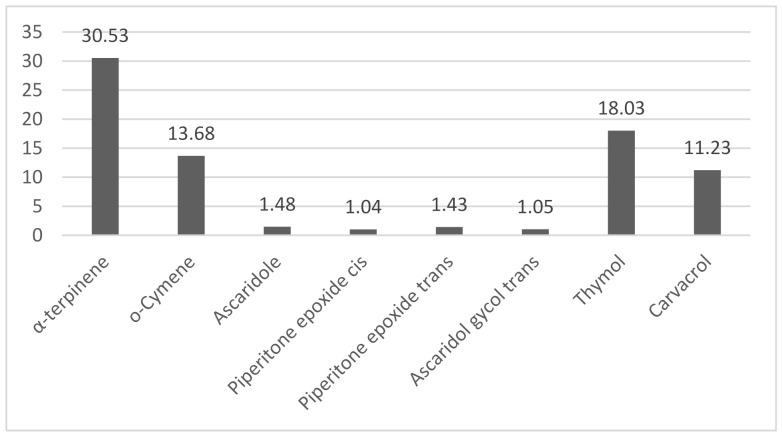
Histogram profile of the *Dysphania ambrosioides* essential oil composition.

The percentage of the identified compounds can be observed in the histogram resulting from the analysis of the chromatographic and spectral data (Figure 1).

### 2.2. Ex Vivo Smooth Muscle Contractility Assay

#### 2.2.1. Effect of the *Dysphania ambrosioides* Essential Oil on K^+^-Induced Contractions Using a *Sus scrofa domesticus* Coronary Artery Model

**Figure 2:**
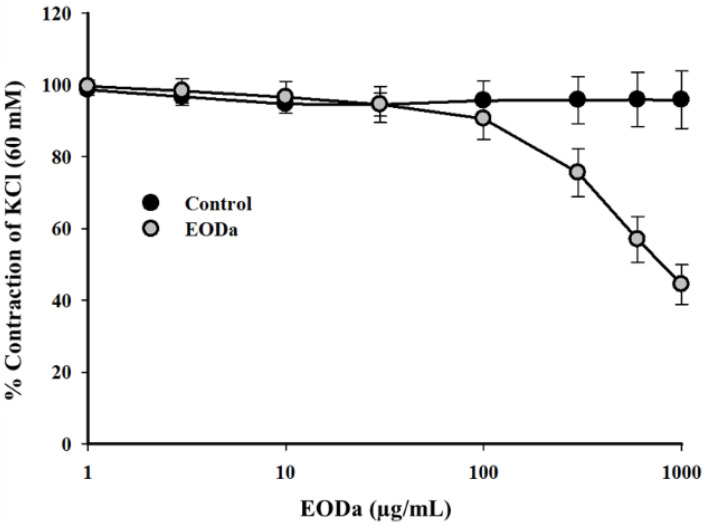
Relaxant effect of the *Dysphania ambrosioides* essential oil (EODa) on contractions maintained by potassium in isolated swine coronary arteries. Representative graph of the relaxant effect of the EODa in contractions maintained by KCl (60 mM) in isolated swine coronary arteries. Values are expressed as the mean ± S.E.M.; *n* represents the number of experiments (*p* < 0.001, one-way ANOVA followed by Holm-Sidak).

Increasing and cumulative concentrations of the EODa (1, 3, 10, 30, 100, 300, 600 and 1000 μg/mL) were administered to evaluate its myorelaxant effect in contractions promoted and sustained by K^+^ in swine coronary artery smooth muscle (electromechanical coupling). Increasing concentrations of the EODa promoted a concentration-dependent relaxation, where its significant effects were observed from the 300 µg/mL concentration (*p* < 0.001, one-way ANOVA followed by Holm-Sidak) (Figure 2).

#### 2.2.2. Effect of the *Dysphania ambrosioides* Essential Oil on Contractions Induced by Serotonin (5-HT) Using a *Sus scrofa domesticus* Coronary Artery Model

**Figure 3:**
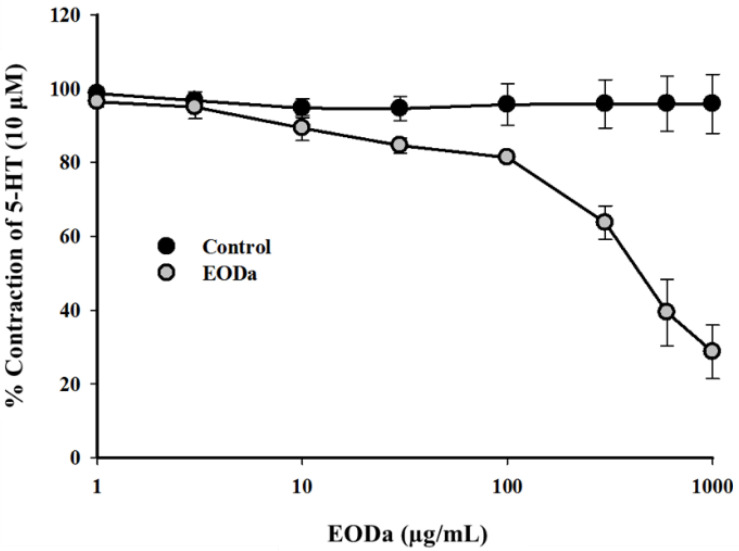
Relaxant effect of the EODa on contractions maintained by serotonin in isolated swine coronary arteries. Representative graph of the relaxant effect of the EODa on contractions maintained by 5-TH in isolated swine coronary arteries. Values are expressed as the mean ± S.E.M.; *n* represents the number of experiments (*p* < 0.005, one-way ANOVA followed by Holm-Sidak).

Increasing and cumulative concentrations of the EODa (1, 3, 10, 30, 100, 300, 600 and 1000 μg/mL) were administered to evaluate its myorelaxant effect on 5-TH-induced contractions in swine smooth muscle coronary arteries (pharmacomechanical coupling). Increasing concentrations of the EODa promoted a concentration-dependent relaxation that was statistically significant from the 30 µg/mL concentration (*p* < 0.005, one-way ANOVA followed by Holm-Sidak) (Figure 3).

The EODa presented a myorelaxant activity in both the electromechanical and pharmacomechanical pathways, however, with a greater efficiency in the pharmacomechanical pathway where contractions were evoked by 5-HT.

### 2.3. D. melanogaster-Alternative Model

#### 2.3.1. Toxicity Assay

The toxicity tests for the *Dysphania ambrosioides* essential oil and α-terpinene against *D. melanogaster* were performed with three concentrations (1, 2 and 4 mg/mL) using the fumigation method. This test uses flies as an alternative model to the use of mammals, generating information for the determination of the toxicity profile of the essential oil and the compound. The calculation for the average lethal concentration (LC_50_) of the EODa was determined as 1.806 mg/mL, by sigmoidal regression, whereas for α-terpinene an EC_50_ of 2.282 was obtained. The aforementioned results were similar to those observed by [25], where the EODa obtained an LC_50_ of 1.96 mg/mL, this being lower than the isolated constituent (Table 1). The same methodology was used to determine the EC_50_ for mobility, for which a value of 1.646 mg/mL was obtained.

**Figure 4:**
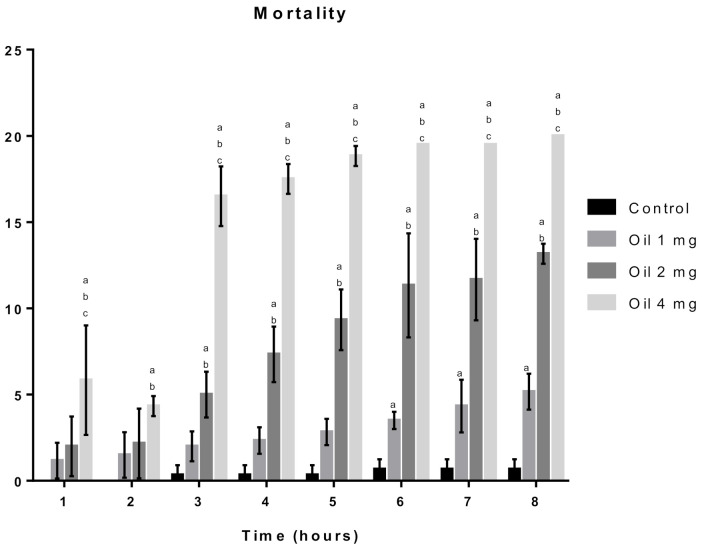
Effect of the *D. ambrosioides* leaf essential oil on *D. melanogaster* survival. Subtitles: Survival was analyzed at the indicated time points. Results are expressed as the Mean ± SD of the number of dead flies after each exposure time. a = *p* < 0.05 compared to the control, b = *p* < 0.05 compared to 1 mg/mL and c = *p* < 0.05 compared to 2 mg/mL.

The EODa showed a significant difference with the 4 mg/mL concentration, when compared to the control and the 1 mg/mL concentration, during all reading times (between 1 and 8 h). From the third hour onwards, a difference between the 2 mg/mL concentration and the control was registered, a fact that was only identified after the sixth hour of reading with the 1 mg/mL concentration, as shown in Figure 4.

**Figure 5:**
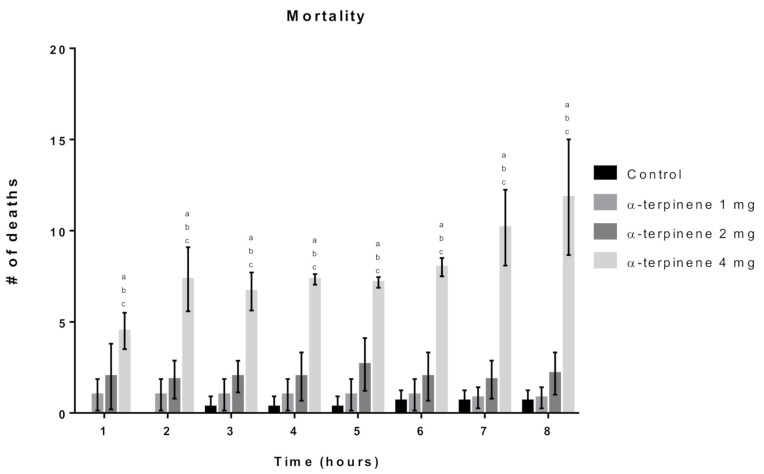
Effect of α-terpinene on D. melanogaster survival. Subtitles: Survival was analyzed at the indicated time points. Results are expressed as the Mean ± SD of the number of dead flies after each exposure time. a = *p* < 0.05 compared to the control, b = *p* < 0.05 compared to 1 mg/mL and c = *p* < 0.05 compared to 2 mg/mL.

The α-terpinene toxicity assays were performed using the following concentrations: 1, 2 and 4 mg/mL. The readings were performed every hour for 8 h, where the 4 mg/mL concentration presented significant differences against all other groups (control, 1 and 2 mg/mL), with this characteristic not being evident in any of the other comparisons, as shown in Figure 5.

The α-terpinene compound present in the *D. ambrosioides* essential oil showed a toxic effect against *D. melanogaster*.

#### 2.3.2. Geotaxis

**Figure 6:**
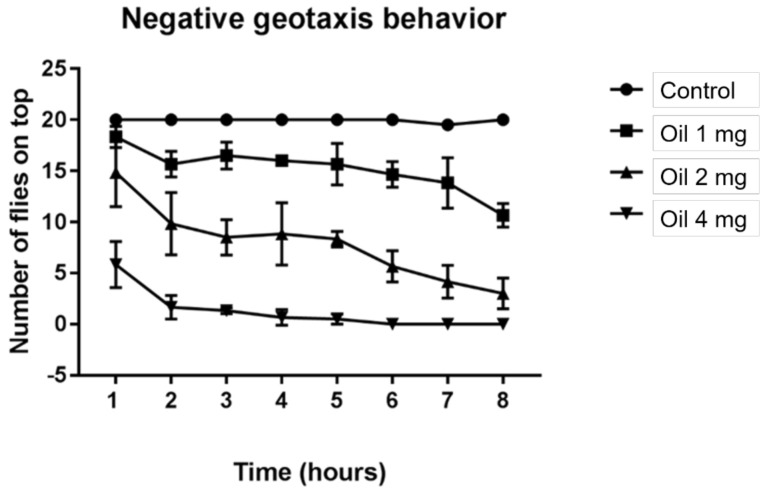
Effect of the *D. ambrosioides* leaf essential oil on the locomotor ability of *D. melanogaster*. Subtitles: Locomotor activity was determined by negative geotaxis behavior. Results are expressed as the mean ± SD of the number of flies able to climb a marked glass column, as previously described, during each exposure time.

Damage to the flies’ locomotor capacity was recorded with all concentrations at all eight recording hours, with the exception of the 1 mg/mL concentration in the first hour, which did not differ statistically from the control. In addition, the 2 mg/mL concentration did not differ significantly from the 4 mg/mL concentration in the last hour (Figure 6).

**Figure 7:**
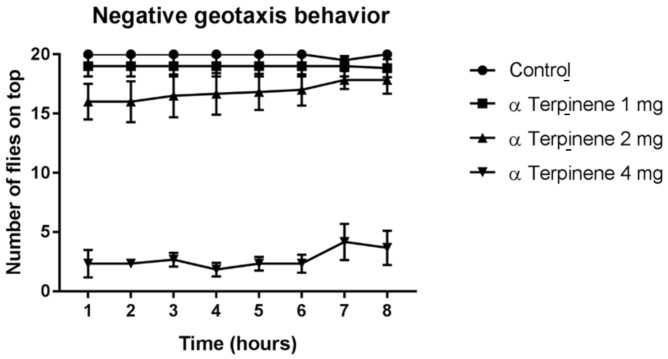
Effect of α-terpinene on *D. melanogaster* locomotor ability. Subtitles: Locomotor activity was determined by negative geotaxis behavior. Results are expressed as the mean ± SD of the number of flies able to climb a marked glass column, as previously described, at each exposure time.

In terms of α-terpinene and insect locomotion, only the 4 mg/mL concentration altered locomotion at all recording times, while the 2 mg/mL concentration also proved to be effective at damaging locomotor capacity up until the sixth hour, after which no differences were observed when compared to the control, thus indicating a reversible action (Figure 7).

**Figure 8:**
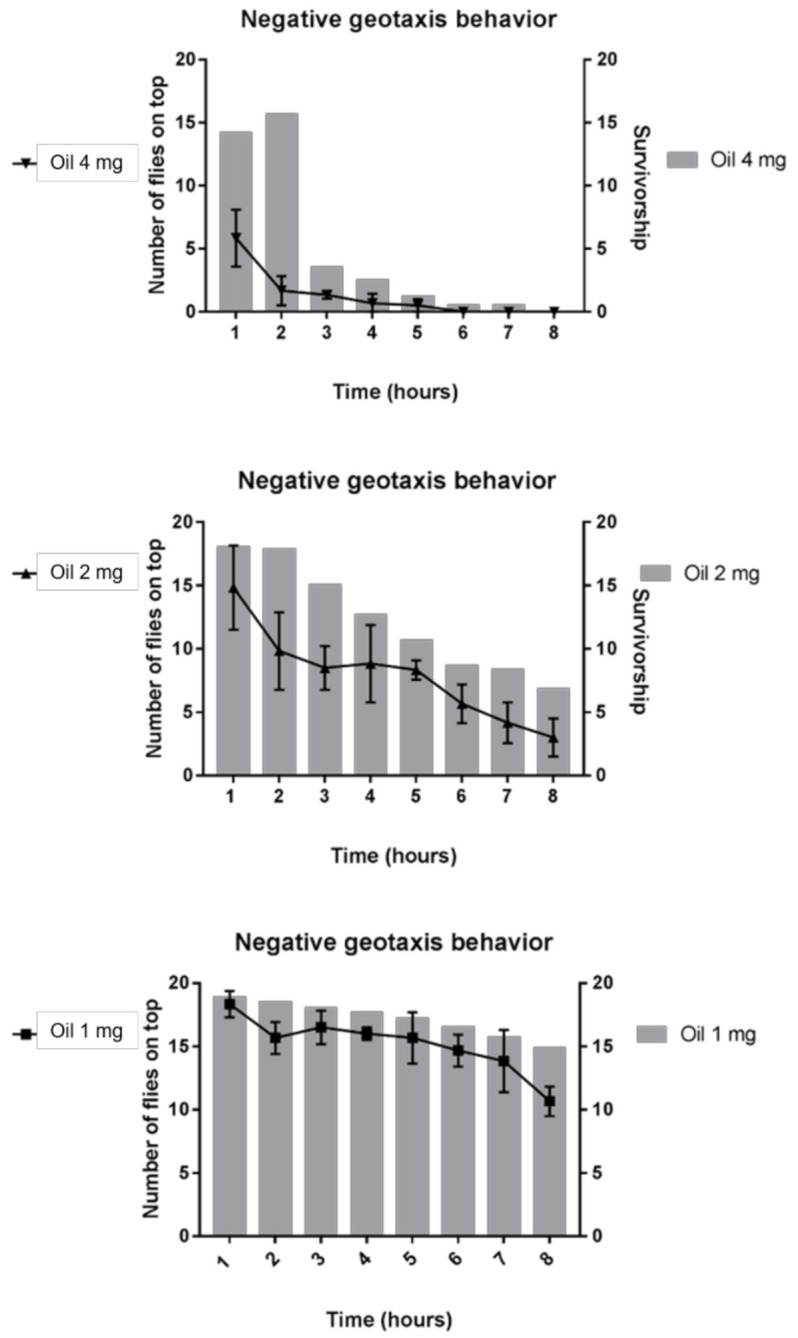
Relationship between alive flies and alterations in mobility.

When superimposing the results from the mortality and negative geotaxis assays, all the tested EODA concentrations (1, 2 and 4 mg/mL) reduced the mobility of the flies that remained alive, a fact that can be well observed in the first hours of exposure to the 2 and 4 mg/mL concentrations, a behavior that was also maintained during the remaining evaluation hours (Figure 8). This section may be divided by subheadings. It should provide a concise and precise description of the experimental results, their interpretation and the experimental conclusions that can be drawn.

## 3. Discussion

The phytochemical profile of the oil studied was established by chromatography and had α-terpinene as a major constituent, with a percentage of 30.53%. The secondary constituents with the highest percentage were: thymol and o-cymene, with 18.11% and 13.68%, respectively. The percentage of the identified compounds can be observed in the histogram resulting from the analysis of the chromatographic and spectral data (Figure 1), and which corroborate with the results presented by [23].

Several studies list the constituents of the EODa, where its composition can be variable, such as in the study by [24], where mainly oxygenated terpenes and sesquiterpenes (α-terpinene 61.04%, 4-carene 13.55% and p-cymene 12.94%) were identified, meanwhile the study by (9) registered cis and trans ascaridol (35.4% and 26.0%, respectively), and p-cymene (29.2%), as the major constituents.

The constituent variations in the aforementioned analyses may be explained by the numerous variables present when comparing studies using the same plant species that have been grown and collected from different locations. These variables include the type of soil, pluviosity indices, wind intensity, collection time and collection season, among other factors that are important influencers in the constitution of essential oils, where such differences have been previously reported [25]. Another factor that must be taken into account is the maturation or age of the plant, which can alter the essential oil composition [26]. Changes in composition can also be observed by chemical reactions that may occur spontaneously following EO extraction, such as by the self-oxidation of α-terpinene, increasing the concentration of several other constituents such as ascaridol and thus reducing its own concentration when in contact with the air [27].

The high potassium concentration generated by the addition of KCl promotes a depolarization of the membrane through a change in electrical charge, which consequently results in the activation of l-type voltage dependent calcium channels (or channels sensitive to dihydropyridine) permitting the entry of Ca^2+^ ions from the external environment into the cytosol. The increase in intracellular Ca^2+^ concentration is the protagonist of excitation–contraction coupling, where this increase in cytosolic calcium concentration mediates the release of calcium from the sarcoplasmic reticulum, further raising calcium ion levels that are available for myosin light chain phosphorylation, one of the final stages of smooth muscle contraction [28,29]. It is noteworthy to highlight that the membrane depolarization caused by potassium also promotes a Ca^2+^- dependent contraction, mediated by the G RhoA protein associated with Rho kinase [30].

Pereira-de-Morais et al. [23] demonstrated that the EODa relaxed the smooth muscle of isolated rat tracheas, with the data indicating that this effect is supposedly due to the blockage of l-type calcium channels. Similarly, Menezes et al. [31] reported a relaxant effect for the *Lippia origanoide* essential oil against contractions promoted by KCL, whilst also indicating the involvement of KCa and KV potassium channels in smooth muscle relaxation, a fact that may explain the relaxation promoted by the EODa since both of these have the same compounds at greater quantities in their composition.

Any effects over K^+^ need to be understood to understand the EO’s mechanism of action, since this ion contributes to the membrane potential in excitable cells, especially neurons, skeletal muscle fiber and smooth muscle fiber, through their action on their channels. On the other hand, EODa effects on serotonin-induced contractions may occur through an external agonist mechanism, where the EODa may bind to membrane receptors and modify the activity of ion channels such as Na^+^ and K^+^ [32,33]. This mechanism begins with the coupling of serotonin to the Gq/11 protein receptor, activating phospholipase C (PLC), which hydrolyzes phosphatidylinositol 4,5-bisphosphate (PIP_2_) into inositol 1,4,5-trisphosphate (IP_3_) and diacylglycerol (DAG). IP_3_ diffuses into the cytoplasm and binds to inositol trisphosphate receptor (IP_3_R receptors), these being calcium channels present in the SR membrane, which when activated release Ca^2+^. Meanwhile DAG together with Ca^2+^ activate PKC that acts in several processes including the regulation of transmembrane Ca^2+^ transport [34]. The observed relaxation may be due, in part, to a blockage of IP_3_R activation by IP_3_, as observed in Pelaia et al., [35], and by other mechanisms such as: increased Ca^2+^-ATPase activity, decreased IP_3_ formation, protein kinase A (PKA) activity and stimulation of the Na^+^/Ca^2+^ exchanger.

Studies with essential oils from aromatic medicinal plants such as *Melissa officinalis* and *Lippia alba* have shown inhibition of contractions promoted by K^+^ and 5-HT (electromechanical and pharmacomechanical pathways) in several organs from rats such as: ileum, trachea, aorta and uterus [6,23,36,37]. Jarvis [38] report the *Lippia alba* essential oil inhibits smooth muscle contractions caused by 5-HT in the trachea and ileum. Assaidi et al. [39] report that the intravenous administration of the aqueous, methanolic and ethyl acetate *Chenopodium ambrosioides* leaf extracts induced arterial hypotension in anesthetized normotensive rats. Thus, results from the present study indicate a relaxant effect for aromatic plants, suggesting the potential *Dysphania ambrosioides* has as a smooth muscle relaxant.

Fruit fly exposure by fumigation to the *Dysphania ambrosioides* essential oil caused a significant increase in mortality. This effect depends on the exposure time and the oil concentration. In the study by Pinho et al. [40], the *Psidium guajava* essential oil presented significant action in terms of *D. melanogaster* mortality, where additionally, the tested concentrations caused a total loss of motor capacity in the flies, depending on the dose and time of exposure. These findings corroborate with the present study and suggest that aromatic plant essential oils may be toxic to arthropods.

The toxic action of the *D. ambrosioides* essential oil has also been investigated in other important models, with studies reporting that this species has pesticidal, larvicidal and insecticidal activity. The *D. ambrosioides* essential oil presented a remarkable in vitro schistosomicidal action, killing 100% of the adult worms in 24 and 72 h, and significantly reducing in vitro cell viability, obtaining however, a selectivity index 31.8 times more toxic to adult *Schistossoma mansoni* worms than the tested cells [41], which suggests a toxic activity with potential applications in pest control.

EODA has also been shown to be toxic to adult houseflies [9] and toxic to *Alphitobius diaperinus* [42]. Therefore, the EODA can be considered in new strategies looking at new possibilities in integrated pest management.

The toxic effect of the EODa may be associated with a change in acetylcholinesterase activity, which in turn is associated with an increase in reactive oxygen and nitrogen species in the neuronal system, which initially would lead to an increase in the activity and concentration of antioxidant enzymes such as catalase and glutathione S-transferase [43,44].

The α-terpinene compound present in the *D. ambrosioides* essential oil showed a toxic effect against *D. melanogaster*. Studies have shown that the toxicity of α-terpinene against *Sitophilus zeamais* adults has LC_50_ values of 5.46 mg L^−1^ [25], this being an important adjuvant component in the composition of other oils used for this purpose [45]. Paes et al. [46] showed that α-terpinene was one of the main compounds identified in the *C. ambrosioides* essential oil, which showed an acaricidal activity. However, the use of essential oil in their entirety have shown greater toxic activity than their major constituents in isolation, thus corroborating with studies that demonstrate the toxicity of selected constituents from mixtures present a synergistic effect between the supposedly active and inactive compounds, and that the presence of all the constituents is necessary for the complete toxicity and/or action of essential oils [45].

The lethal effect of terpenes and/or essential oils is the result of the toxic action of these compounds on various receptor targets, especially in signaling or regulatory pathways that are directly associated with the nervous system, such as: acetylcholinesterase interference, binding to oxytocin, nicotinic, GABAergic and tyramine receptors [47]. In addition to numerous target effects, where these substances do not act on a single target, the complex composition of essential oils contribute to the lethality of insects, not only because of their pharmacodynamic effect, but also because of the possibility of activating on several targets, and pharmacokinetic effects [48], where the complex itself may contribute to better absorption, thus greater bioavailability of active compounds, resulting in the competition of metabolic pathways by the EO constituents, among many other variations, which may occur in this process [49].

It has been suggested that the reduced mobility of *D. melanogaster*, observed by the lower number of flies reaching the top of the column, is a result of the effective inhibition of acetylcholinesterase (AChE) [50,51]. Thus, we may infer the EO’s ability to induce relaxation in smooth muscle may be explained by an increased concentration of acetylcholine in the vascular endothelium. Acetylcholine in the endothelium acts through muscarinic receptors coupled to G proteins, which contrary to stimulating calcium influx for an effective contraction, induces the activation of nitric oxide synthase promoting the production of nitric oxide and relaxation [52,53,54,55]. However, it is noteworthy that other pathways causing changes in the mobility of flies may be involved, including the dopaminergic pathway, since decreases in dopamine significantly alter insect mobility [56].

The α-terpinene was one of the main compounds identified in the *D. ambrosioides* essential oil, also known as *Chenopodium ambrosioides*, which showed acaricidal activity [46]. Moreover, α-terpinene has shown an acetylcholinesterase inhibitory activity [57], thus corroborating with the findings in this study, since α-terpinene is the main monoterpene of present in the EODa.

The greatest damage to locomotor capacity was recorded when comparing the EODa to its isolated major constituent, a fact that consolidates the observation that toxic effects are more potent when a complex mixture is tested rather than a single constituent.

Several studies report that decreased acetylcholinesterase activity is strongly associated with decreased mobility [58,59,60] or permanent locomotor disability [61]. The comparison between live and mobile flies shows a direct negative correlation, both in terms of the EODa concentration and the exposure time, which suggests that locomotor disability precedes general toxic effects, which may lead to the death of the insect, where this result may be explained by the action of essential oil constituents on different target enzymes and genes [50,62].

## 4. Materials and Methods

### 4.1. Plant Collection and Identification

The *Dysphania ambrosioides* (L.), Mosyakin and Clemants, botanical material was collected from the Botanical Garden of the Natural Products Research Laboratory (Horto Botânico do Laboratório de Pesquisa de Produtos Naturais—LPPN), at the Regional University of Cariri (Universidade Regional do Cariri—URCA, Crato, Brazil) (coordinates: 07°14′19.2″ latitude S. and 39°24′52.8″ longitude W.). The species was identified by Prof. Dr. Maria Arlene Pessoa da Silva as belonging to the *Dysphania ambrosioides* (L.), Mosyakin and Clemants, species, from the Chenopodiaceae family. An exsiccate was deposited at the Caririense Dárdano de Andrade-Lima Herbarium (Herbário Caririense Dárdano de Andrade-Lima, Crato, Brazil), from the Regional University of Cariri (Universidade Regional Cariri—URCA, Crato, Brazil), under voucher # 12.208.

### 4.2. Essential Oil Extraction

The leaves from the plants were collected and cut into approximately 1 cm^2^ pieces. Subsequently, the plant material was immersed in distilled water and subjected to extraction by hydrodistillation in a Clevenger type apparatus to obtain the essential oil. The extractions were performed in triplicates and treated with anhydrous sodium sulfate. The oil was then filtered through cotton and transferred with a Pasteur pipette to an amber glass and stored at −20 °C. The extraction and analysis procedures were performed at the Natural Products Research Laboratory (Laboratório de Pesquisa de Produtos Naturais—LPPN)-Regional University of Cariri (Universidade Regional do Cariri—URCA, Crato, Brazil).

### 4.3. Phytochemical Analysis

The essential oil was subjected to gas chromatography with an HP-5 non-polar column (Agilent J&W, Santa Clara, CA, USA, 60 m × 0.25 mm internal diameter; film thickness 0.25 μm) and coupled to a mass spectrometer with an Agilent 5975C Series quadrupole analyzer (Agilent Technologies, Palo Alto, CA, USA). The conditions of analysis were as follows: carrier gas helium with constant flow of 1 mL per min and for injection of the sample initial temperature of 40 °C (maintained for 2 min), heating ramp of 4 °C/min and final temperature of 230 °C, which was maintained for 5 min. The compounds eluted from the chromatographic column were ionized by electron impact at 70 eV. The ionization source was maintained at 230 °C and the quadrupole at 150 °C. The compounds were identified by analyzing the mass spectra of each chromatographic peak, comparing with authentic standards and calculating retention indices. The calculated values were compared with the retention rates published in the literature [23].

The same solution was quantified by injection in triplicate of 1 μL in the splitless mode of this solution in a gas chromatograph (Thermo trace GC ultra) equipped with non-polar column VB-5 (60 m × 0.25 mm internal diameter; film thickness 0.25 μm). The samples were analyzed under the following conditions: initial temperature of 40 °C (maintained for 2 min), heating ramp of 4 °C/min and final temperature of 230 °C, which was maintained for 5 min. The carrier gas used was nitrogen, at a constant flow of 1 mL per minute and the injector temperature was maintained at 250 °C. The compounds eluted from the chromatographic column were detected using flame burners (FID) at 250 °C.

### 4.4. Ex Vivo Assays and Pharmacological Tests

According to the Normative Resolution Concea *n*° 30, on 2 February 2016, cadavers and their parts, originating from the activities of slaughterhouses, butchers or rural producers for consumption, are exempt from going through the Commission for Animal Use and Experimentation (Comissão de uso e Experimentação Animal—CEUA).

Adult swine (*Sus scrofa domesticus*) coronary arteries were obtained from a local slaughterhouse (Frigorífico Industrial do Carir—Leandro Bezerra located at Av. Paulo Maia *n*° 2000, São José district, Juazeiro do Norte—Ceará, Brazil).

During transport to the laboratory, the coronaries were placed in a modified Tyrode (TM or Tyrode) nutrient solution at 4 °C with the following composition in mM: (136.0 NaCl; 5.0 KCl; 0.98 MgCl_2_; 0.36 NaH_2_PO_4_; 11.9 NaHCO_3_; 2.0 CaCl_2_ and 5.5 glucose). The pH was adjusted to 7.4 with 1 M HCl and/or 1 M NaOH. The coronaries were dissected and sectioned into rings measuring between 4 and 5 mm in length. The coronary rings were kept in an isolated organ bath with a 10 mL capacity for the Tyrode nutrient solution, maintained under continuous aeration by air bubbling and a temperature of 37 °C. The contractile activity measurements for the tissues were recorded using a rod connected to a force transducer (TRI, model 210, Panlab, Spain), coupled to a differential amplifier (DATAQ, model PM-1000, Akron, OH, USA), with input to a digital analog converter board (DATAQ DI-200) installed on a computer, whose collected data were converted into traces and stored into files using the WINDAQ software (DATAQ Instruments, Inc. Akron, OH, USA). The isolated coronary rings were subjected to a 1 gf (gram force) tension and acclimatized for a period of 1 h. All protocols started with two subsequent contractions reproduced by the addition of 60 mM KCl (K60) to the studied coronary rings, in a hypertonic manner, where after reaching stable values, a plateau, the maximum response obtained was considered the maximum contraction of the ring. Only experiments with reproducible contractions were considered viable for the experimental series.

All experiments were performed in triplicates, accompanied by their control, which was subjected to the same conditions and the same experimental protocols. The control preparations received only the vehicle, Tween, diluted in Tyrode’s solution, in the same proportions used in the experimental preparations.

### 4.5. Toxicity Test with the Drosophila melanogaster Alternative Model

#### 4.5.1. Drosophila Stock and Culture

*D. melanogaster* (Harwich strain) flies were obtained from the National Species Stock Center, Bowling Green, OH. The flies were raised in 330 mL glass bottles (15 cm in height and 6.5 cm in diameter), cultured with a medium containing: (83% corn mass, 4% sugar, 4% lyophilized milk, 4% soy bran, 4% wheat bran and 1% salt), 1 g of Nipagin (Methylparaben) and 1 mL of a solution containing *Saccharomyces cerevisiae*. The flies were kept at a temperature of 25 °C with a relative humidity of 60% [23]. All experiments were performed with the same strain.

#### 4.5.2. *D. melanogaster* Assays and Essential Oil Exposure

Fly exposure to the essential oil was performed using the fumigation protocol as described: 20 adult flies (male and female) were placed in 130 cm^3^ flasks, containing a filter paper soaked with 20% sucrose in distilled water at the bottom. A filter paper (1 cm^2^) was attached to the inside of the bottle’s screw cap to apply different doses of the essential oil. In doing so, the flies feed on the sucrose solution at the bottom of the flasks while the essential oil is allowed to volatilize from the top and reached the fly’s respiratory system. The bottles received the following treatments: 1% sucrose (control) and 1, 2 or 4 μg/mL of the essential oil or the tested constituent. Fly survival readings were taken every hour until the eighth hour. The results are shown as the number of live flies (mean ± SD) obtained from six independent experiments.

#### 4.5.3. Locomotor Assay

Locomotor capacity was evaluated following the negative geotaxis behavior as described by S. Boutkhil et al. [24], with some modifications. Twenty adult flies (1–4 days old, males and females) were exposed to the essential oil as described in the previous section. Briefly, after the hourly fly mortality count, a negative geotaxis test was performed simultaneously with the surviving flies, where this consisted of counting the number of flies that rise above 5 cm in the glass column of the experiment itself, during a 5 s time interval. The tests were repeated five times with 1 min intervals. The results are presented as the number of flies that reached the top ± SD obtained from three independent experiments.

### 4.6. Statistical Analysis

Data are expressed as the mean ±S.E.M. For statistical analysis and graph production, the Sigma Plot 11.0 software was used for the ex vivo assays using a one-way ANOVA followed by Holm-Sidak, and the GraphPad Prism 7.0 software for the other tests. Results considered statistically significant obtained a null hypothesis probability of less than 5% (*p* < 0.05). Student’s *t*-tests and analysis of variance (two-way ANOVA), followed by Tukey’s *t*-test were used. For calculating the EC_50_ values, logarithmic interpolation and sigmoidal regression were performed, with the concentration capable of producing 50% of the maximum effect being considered as the EC_50_, with the calculations being performed for each experiment.

## 5. Conclusions

The *Sus scrofa domesticus* coronary artery ex vivo muscle contractility methodology has shown the relaxant activity of both the EODa and its major compound on smooth muscle contractions induced by potassium and serotonin. The EODa composition revealed the presence of oxygenated terpenes, which are capable of promoting toxic effects in insects from a low concentration, in addition to inducing a precocious impairment in fly mobility, which creates a difficulty in dispersing these insects. The above makes the EODa a potential product for carrying out necessary studies on the safety of these compounds for humans, both for its myorelaxant action on the vascular system, and its toxicity for cultivating crops, with the possibility of developing formulations with more effective, safe and attractive cost benefits.

## Figures and Tables

**Table 1:** Fumigant toxicity of the *Dysphania ambrosioides* essential oil and α-terpinene against *D. melanogaster* adult flies.

Compound	LC_50_ (mg L^−1^ Air)	95% Fiducial Limits
EODa	1.806	1.588–2.296
α-Terpinene	2.282	1.933–2.671

## Data Availability

Not applicable.

## Sample Availability

Not available.
